# Exploring the Receptivity and Feasibility of Just-In-Time Support for Healthy Food Choices: Mixed-Method Insights for Adaptive Intervention Development^☆^

**DOI:** 10.1016/j.cdnut.2026.109391

**Published:** 2026-06-06

**Authors:** Laura HH Winkens, Nieke A Sonneveld, Arend Ligtenberg, Ayla Schwarz, Monique Simons

**Affiliations:** 1 Chair Group Consumption and Healthy Lifestyles, Wageningen University & Research, Wageningen, The Netherlands; 2 Laboratory of Geo-Information and Remote Sensing, Wageningen University and Research, Wageningen, The Netherlands

**Keywords:** Just-in-Time, context-aware, health behavior change, geofencing, eating behavior, healthy lifestyle, mHealth

## Abstract

**Background:**

A suboptimal diet is a major health risk, yet adopting and maintaining healthy eating habits remains challenging. Just-in-Time (JIT) digital support may help, however, little is known about when and where users are most receptive to such support.

**Objectives:**

This study aimed to evaluate receptivity to a researcher-developed JIT-based app that prompts healthy food choices near food outlets and at preset times. The primary outcome was receptivity to JIT prompts, defined as users’ willingness and ability to receive, process, and act upon prompts in daily life. Exploratory outcomes included usability, perceived privacy and perceived effectiveness.

**Methods:**

In a single-arm mixed-methods study, 14 adults (M = 27 y) used the app for 1 wk after selecting a nutritional health goal. Data were collected via poststudy questionnaires, in-app feedback, and interviews with 8 participants. Receptivity was assessed based on participants’ willingness and ability to engage with prompts in real-world contexts. Quantitative data were analyzed descriptively and using exploratory statistical tests; qualitative data were analyzed thematically.

**Results:**

Receptivity to JIT prompts was highest when prompts were delivered at meaningful times and locations, particularly at home and in supermarkets, and appeared higher during more positive and calmer emotional states. Exploratory feasibility findings indicated that usability was affected by technical issues and battery drain. Participants generally expressed willingness to share personal data when used transparently for personalization. Users reported improvements in self-rated diet quality and goal-related dietary behavior, and emotional states were associated with perceived momentary effectiveness. Lower-educated participants reported more installation difficulties, less favorable perceptions of prompt tone, and smaller improvements in food choices.

**Conclusions:**

Our findings provide initial insight into when and where individuals are most receptive to JIT support for healthy eating, informing the design of future adaptive dietary interventions tailored to users’ contexts and momentary states.

This trial was registered at clinicaltrials.gov as NCT05773625 (https://clinicaltrials.gov/ct2/show/NCT05773625).

## Introduction

Suboptimal diets significantly contribute to the onset of chronic diseases such as cardiovascular disease, cancer, and type 2 diabetes [1], and account for a substantial proportion of disability-adjusted life years and premature mortality [2]. To mitigate these health risks, interventions that promote dietary behavior change are urgently needed. Recent modeling work shows that adhering to dietary guidelines could substantially reduce the future burden of noncommunicable diseases in the Netherlands [3]. For example, eliminating processed meat could reduce the type 2 diabetes incidence cases by up to 24.5% in men and increase disease-free life expectancy by up to 2.5 y [3]. These findings underscore the need for strategies that support healthy dietary behaviors in daily life.

Digital health technologies, particularly smartphone apps, have become popular tools for promoting healthy lifestyles. However, their long-term effectiveness often diminishes [4], partly because lifestyle behaviors are shaped by multiple, dynamic, and personal factors such as mood, the physical environment, and social situations [5]. This complexity calls for interventions that are tailored to the individual and their context [5,6]. Advances in real-time health measurements present promising opportunities to deliver timely, context-specific support at moments when individuals are most likely to act on health-related decisions [7].

One promising approach is to apply “Just-in-Time” (JIT) principles, which aim to provide the right type of support at moments when individuals are most receptive and able to act. In the context of healthy eating, this could mean providing support at key decision points, such as grocery shopping, meal preparation, or eating occasions. Although JIT support has shown promise in areas such as physical activity and smoking cessation [8,9], applications to dietary intake remain limited [10,11]. As a result, there is limited empirical evidence when, where, and for whom such support is most acceptable and relevant. Addressing these questions is essential before developing and scaling up fully adaptive interventions [12,13].

To address this gap, the primary goal of this study was to assess user receptivity to the *EetWijzer* app, a smartphone-based intervention developed by our research team in co-creation [14,15]. The app is inspired by JIT principles and provides time- and location-based prompts using location-based service techniques [16] to support healthy food decisions during key moments such as grocery shopping or meal occasions [[17], [18], [19]]. Receptivity is defined as individuals’ transient willingness and ability to receive, process, and use JIT support [20] (see Table 1 [[20], [21], [22], [23]] for definitions). Specifically, we addressed the following research question: When and where are users most receptive to receiving JIT support for healthy eating?

**TABLE 1:** Definitions of receptivity and exploratory feasibility outcomes

Concept	Definition
Receptivity	*“The individual’s transient ability and/or willingness to receive, process, and utilize* Just-in-Time *support”* [20]
Usability	*“The ease with which users can use a technological application to reach a particular goal”* [21]
Privacy	Information privacy encompasses “*an individual's ability to personally control the collection, use, and proliferation of information about herself”* [22]
Perceived effectiveness	*“The subjective impact as experienced or reported by …., rather than objectively assessed outcomes of the interventions”* [23]

Feasibility outcomes (usability and relevance, perceived privacy, and perceived effectiveness) were examined exploratorily. We also explored whether educational levels were associated with these feasibility indicators. This study informs future development of adaptive, fully JIT interventions tailored to users’ needs and contexts.

## Methods

### Study design and procedure

This study employed a 2-wk feasibility design with pre- and postmeasurements to evaluate the feasibility of the *EetWijzer* app. The study was conducted between January and May 2023.

#### Pre-study

Participants were first provided with an information letter and gave signed informed consent. They then received guidance on how to install and use the *EetWijzer* app. Following successful installation, participants completed a pre-study questionnaire.

#### Intervention period

The study was divided into 2 distinct but consecutive weeks: a control week and an intervention week. During the control week, the app tracked participants’ locations without sending any intervention prompts. In the subsequent intervention week, the app continued to track locations but also sent prompts related to the participants’ chosen health goals and collected responses to these prompts.

#### Poststudy

At the end of the study, participants completed a poststudy questionnaire. Those who agreed were invited for a 20-min online interview. Participants received a €50 voucher for completing the study and an additional €10 voucher for participating in the interview.

The study received approval from the Social Ethics Committee of Wageningen University. Ethical approval was deemed unnecessary by the Medical-Ethical Committee Nederland-Oost (2022-13712). The study was registered retrospectively at the clinicaltrials.gov (NCT05773625) on 6 March, 2023 (first participant enrolled on 12 January, 2023) because the authors did not receive a response to their original registration submission (12 January, 2023) to the Clinical Trial Registry.

### Sample size

As this study aimed to assess feasibility rather than effectiveness, no formal power calculation was performed. The target sample size was 20 participants and was determined pragmatically based on available resources, anticipated recruitment capacity, and the study timeframe.

### Participants

Participants were recruited through a Facebook campaign targeting residents of Wageningen aged 18 to 65 y. Additional recruitment was conducted via flyers distributed in community centers, welfare organizations, the city library, general practices, and pharmacies. The flyers specifically included the question: “Do you have difficulty accessing and using health information?” This was done to reach individuals with low health literacy. This focus was intentional, as individuals with low health literacy often experience poorer health outcomes and higher healthcare costs. The goal was to evaluate the app’s feasibility and effectiveness for all participants while specifically including this group who may benefit most from tailored and timely support.

To be included in the study, participants had to reside in Wageningen, be ≥18 y, speak Dutch, own an Android phone, and be open to improving their eating behavior.

### App functionality

**FIGURE 1:**
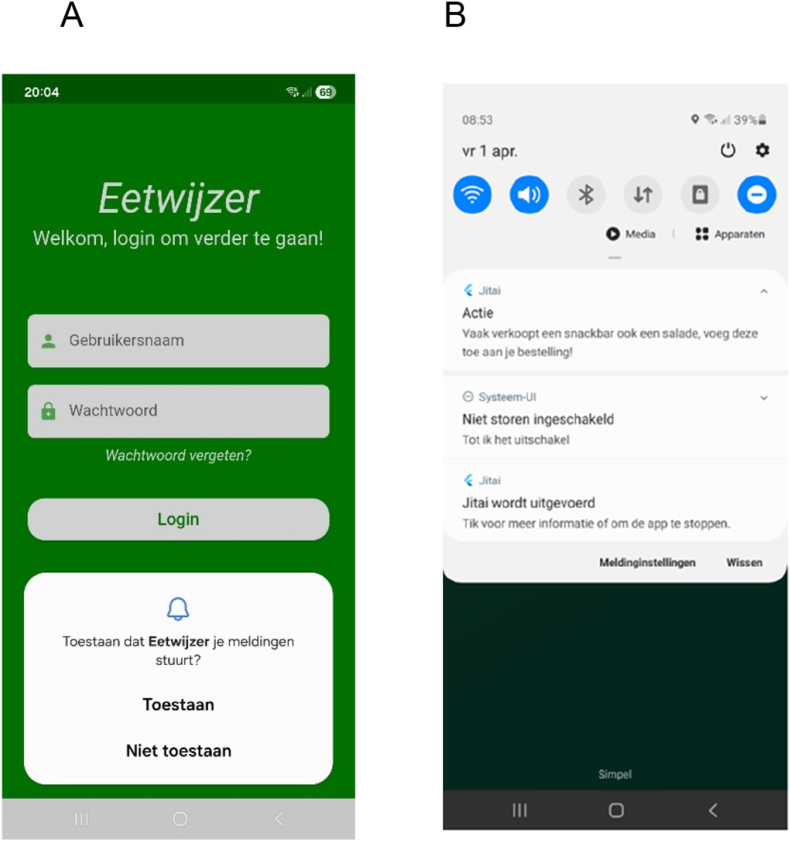
EetWijzer app interface. (A) Login screen. (B) Example of a Just-in-Time prompt delivered in a real-world context, i.e., a fast-food outlet.

The *EetWijzer* app was developed collaboratively with potential end-users from diverse socioeconomic backgrounds and is available for Android devices via the Google Play Store. See Figure 1 for an impression of the *EetWijzer* interface.

After installation, users create an account and select 1 of 3 behavioral goals: healthier snacking, eating less meat, or increasing fruit and vegetable intake. Once activated, the app tracks users’ locations using a Geographic Information System. When users enter a predefined intervention area (Wageningen, where this study was conducted), the app sends proximity-based prompts if they remain within a specific radius of a food outlet for a defined period.

The app’s proximity-based prompts were triggered when participants approached specific types of food outlets, such as convenience stores, restaurants, and cafes. We used a manual mapping approach during development to define these locations and their notification radius. The research team physically walked through the study area, identifying key outlets based on local knowledge, visible landmarks, and the types of products sold. This method allowed us to fine-tune detection accuracy and ensure that notifications were triggered appropriately and relevant to participants’ behavioral goals.

Additionally, time-based prompts were strategically designed to align with users’ daily routines and specific goals. For instance, prompts related to healthier snacking were sent at 11:00, 15:00, and 20:00, whereas prompts for eating less meat or increasing fruit and vegetable intake were delivered at 12:00 and 17:00. These timings are intended to provide reminders at moments when users are likely to be making food-related decisions.

### Behavior change strategies

Digital interventions are increasingly recognized as effective tools for supporting dietary behavior change, particularly when they incorporate well-established behavior change techniques such as goal setting, action planning, and prompts/cues [24]. The *EetWijzer* app is designed around these principles by delivering real-time prompts aligned with each user’s dietary goal.

Building on goal-setting theory [[25], [26], [27]], the app provides users with a concrete target to work toward [28]. The app complements this with JIT prompts, triggered by location (via GPS) and time of day, to offer actionable support in relevant decision-making contexts. The *EetWijzer* app tailor notifications based on each user’s selected dietary goal, delivering contextually relevant prompts in real-time. These in-app messages are designed to keep goals top of mind and offer immediate support during key decision-making moments [29]. In addition, the content of each prompt is positively framed, provides practical suggestions (e.g., food swaps or recipes), and follows best practices for effective, personalized health communication [[30], [31], [32]]. By delivering timely, tailored support, the app aims to enhance users’ motivation and engagement throughout their behavior change journey [33,34]. Example prompts are included in Supplemental Table 1.

From a theoretical perspective, this approach also draws on the Fogg Behavior Model, which emphasizes that behavior occurs when motivation, ability, and a prompt coincide [33]. Even in moments of low motivation or decision fatigue, well-timed, low-effort suggestions can support behavior. This is particularly important for dietary decisions, which are often habitual and influenced by contextual cues. By reducing the cognitive burden of planning and decision-making, the app addresses decision fatigue and facilitates goal-aligned behaviors.

Although the app does not collect real-time dietary intake or behavior data (e.g., via self-monitoring or passive sensors), it does deliver prompts that are closely aligned with users’ goals and delivered in moments of opportunity. This aligns with Feedback Intervention Theory [35], which highlights the importance of directing attention to the behavioral task at hand, and is consistent with the design principles of JIT-based interventions [20,36].

In JIT interventions, actionable feedback refers to specific, context-aware guidance that supports users in deciding *what* action to take and *when* and *where* to take it [37], for example through reminders near food outlets or suggestions at habitual snacking moments. This conceptualization is consistent with prior work describing JIT messages as behavioral triggers delivered at critical moments [20,36]. It also aligns with findings from our co-creation study (i.e., focus groups, survey study, and beta study [14]) and earlier literature [38], which identified reminders of daily health goals and nearby healthy food options as key end-user preferences. Although *EetWijzer* does not provide direct performance feedback, its prompts still offer meaningful support by reinforcing users’ intentions, increasing awareness of health goals, and nudging healthier choices in relevant moments.

### Outcomes

The study outcomes were pre-registered on clinicaltrials.gov. The primary outcome of this study was receptivity (Table 1). Secondary outcomes specified in the study registration (movement patterns) are not reported in the present article and will be addressed in future publications. Exploratory outcomes included usability, relevance, perceived privacy and perceived effectiveness.

### Measurements

#### Participant characteristics

The pre-study questionnaire assessed demographics (age, sex, and educational level), self-rated health and diet quality, and an initial indicator of health literacy.

*Educational* *level* was categorized according to the International Standard Classification of Education used by Statistics Netherlands (CBS) [39] into: low (primary and lower secondary education including prevocational secondary education and the first years of senior general secondary education); medium (upper secondary education or basic vocational training which is vocational training for middle management or specialist education); and high (higher education, including Bachelor’s, Master’s, or doctoral programs).

Participants rated *perceived health*, *diet quality*, and *goal-related diet quality* (e.g., healthiness of snack intake) on a 5-point scale ranging from poor to excellent. Self-rated diet quality was assessed with the question “In general, how healthy is your overall diet?,” a validated proxy for more comprehensive diet quality measures [40].

An initial indication of *health literacy* was obtained by asking: “Do you have trouble finding, understanding, and using health information?” with response options “yes, always,” “yes, sometimes,” and “no, never” [41].

In the poststudy questionnaire, *health literacy* was measured using the Dutch translation of the European Health Literacy Survey Questionnaire short form (HLS-EU-Q16) [42], a validated 16-item instrument assessing self-reported difficulties in accessing, understanding, appraising, and applying information to tasks related to making decisions in health care, disease prevention, and health promotion contexts. The HLS-EU-Q16 was obtained from the HLS-EU-Q47 using Rasch Analysis [41]. Each item was rated on a 4-point Likert scale (very difficult, difficult, easy, and very easy) plus “don’t know/no answer.” Following the authors’ scoring procedure, “very difficult” and “difficult” were coded as 0, and “easy” and “very easy” as 1. The total summed score (0–16) was categorized into 3 levels: inadequate (0–8), problematic (9–12), and sufficient (13–16).

#### Primary outcome: receptivity

Receptivity to JIT prompts was assessed using a multimethod approach capturing real-time in-app responses and retrospective evaluations of openness to prompts in daily life through questionnaires and interviews.

#### In-app measures

During the intervention period, participants received prompts in real-world contexts. After each prompt, they indicated whether they were open to the prompt at the given location (yes/no) and time (yes/no). They also reported their current emotional state using the 7 universal basic emotions, namely: happiness, relaxation, disgust, fear, anger, surprise, and sadness [43,44] and additionally stress, given its relevance to eating behavior [45,46].

### Poststudy questionnaire

See Supplemental Methods 1 for all questionnaire items. To further assess receptivity, participants indicated at which locations (e.g., supermarkets, at home), times of day (e.g., morning), and days (e.g., weekdays), receiving prompts was most or least convenient. They also reported during which emotional states they preferred to receive prompts, using the same 8 emotion categories as in the in-app assessment.

### Qualitative interviews

Semi-structured interviews further explored participants’ experiences and perceptions of receiving prompts, including contextual factors influencing receptivity (see Qualitative interviews below).

### Exploratory outcomes

Exploratory outcomes were also assessed using poststudy questionnaires, in-app measures, and interview data.

#### Poststudy questionnaire

Questionnaire items are provided in Supplemental Methods 1.

*Relevance and usability* were assessed with 6 statements addressing notification tone, relevance, fit with daily routine, ease of installation, ease of use, and time burden. Items were rated on a 5-point Likert scale ranging from completely disagree to completely agree. Participants were also asked whether they would continue using the app, recommend it to others, and provide additional comments about their experiences.

*Perceived privacy* was assessed with 1 item evaluating perceived privacy during app use, rated on a 5-point Likert scale ranging from completely disagree to completely agree.

*Perceived effectiveness* was assessed using 3 statements addressing goal attainment, healthy eating knowledge, and conscious food choices. Items were rated on a 5-point Likert scale ranging from completely disagree to completely agree. Participants also re-rated their overall diet quality and goal-related diet quality on a 5-point scale ranging from poor to excellent to assess perceived changes in dietary behavior.

#### In-app measures

For usability and relevance, participants completed daily reflection questions about their experiences with the prompts that day. They rated the number of prompts received (5-point scale from way too little to way too much), indicated whether they would have liked to receive a prompt at an unprompted moment (and specified when/where), and answered 2 open-ended questions about which prompts they liked or disliked and why. Participants could also describe any improvements they noticed or would suggest for the app.

For perceived effectiveness, after each prompt, participants rated perceived contribution of the prompt to their health goal (5-point scale from very unlikely to very likely).

#### Qualitative interviews

Participants who indicated willingness to participate in a follow-up interview were contacted to schedule a 20-min online interview via Microsoft Teams. The interviews aimed to elaborate on participants’ experiences using the app and to contextualize findings from the questionnaires and in-app data.

An interview guide (Supplemental Methods 2) and coding framework were developed around receptivity and the 3 feasibility domains: usability and relevance, perceived privacy, and perceived effectiveness. Interviews were conducted by a single researcher (NS) and began with a brief introduction to ensure participants understood the study’s aims and procedures. All interviews were audio recorded with participants’ consent.

### Statistical analysis

All quantitative analyses were conducted using IBM SPSS Statistics version 28.

Statistical significance was set at *P* < 0.05 for all analyses.

### Sample characteristics and dropout analysis

Descriptive statistics were computed for all variables. Baseline characteristics were compared between participants who completed the study and those who dropped out. Given the small sample size and non-normal distribution of the ordinal variables, as confirmed by Shapiro–Wilk tests and visual inspection, nonparametric tests were used: chi-square tests for categorical variables (sex) and Mann–Whitney U tests for continuous variables (age, overall self-reported health, and healthiness of the eating pattern).

### Questionnaire data

Descriptive statistics were calculated for all items from the pre- and poststudy questionnaires. For the exploratory feasibility aspects (usability and relevance, perceived privacy, and perceived effectiveness), mean and median scores were calculated to summarize user evaluations of the *EetWijzer* app. To assess changes in perceived dietary behavior, Wilcoxon signed-rank tests were used to compare self-reported overall diet quality and goal-related diet quality between the pre- and poststudy assessments.

### In-app data

Descriptive statistics were computed for all in-app responses related to prompts (openness to receiving prompts, emotions, and perceived goal relevance). To explore factors associated with perceived goal relevance (exploratory analysis), linear mixed-effects models were fitted with participant ID as a random intercept to account for repeated observations and openness to location, openness to time, and emotion as categorical fixed effects. Location and timing were binary self-reported contextual indicators reflecting whether participants considered the current context suitable for receiving a prompt (0 = no, 1 = yes). Emotion was included as a categorical variable reflecting the participant’s reported emotional state at the time of the prompt.

### Differences by educational level and health literacy

To explore potential differences in feasibility evaluations between subgroups, Mann–Whitney U tests were conducted to compare responses on the poststudy questionnaire between high compared with medium educational levels, and sufficient compared with problematic/inadequate health literacy levels based on the HLS-EU-Q16 classification. Analyses focused on usability, perceived effectiveness, and perceived privacy scores.

### Qualitative analysis

Interviews were recorded and automatically transcribed verbatim using Microsoft Teams, then reviewed and edited into nonverbatim transcripts by the interviewer (NS). Data were analyzed using thematic analysis [47] in Atlas.ti to identify recurring patterns of meaning. A coding framework was developed based on receptivity and the 3 predefined feasibility factors: usability and relevance, perceived privacy, and perceived effectiveness. The framework was applied deductively while new codes emerging from the data were added inductively. One researcher (NS) coded all interviews, and a second researcher (AS) independently double-coded 3 transcripts (38%) to ensure reliability. Discrepancies were resolved through discussion until consensus was reached, and the coding framework was iteratively refined during this process. Final themes were used to structure the qualitative results.

## Results

### Participants

**FIGURE 2:**
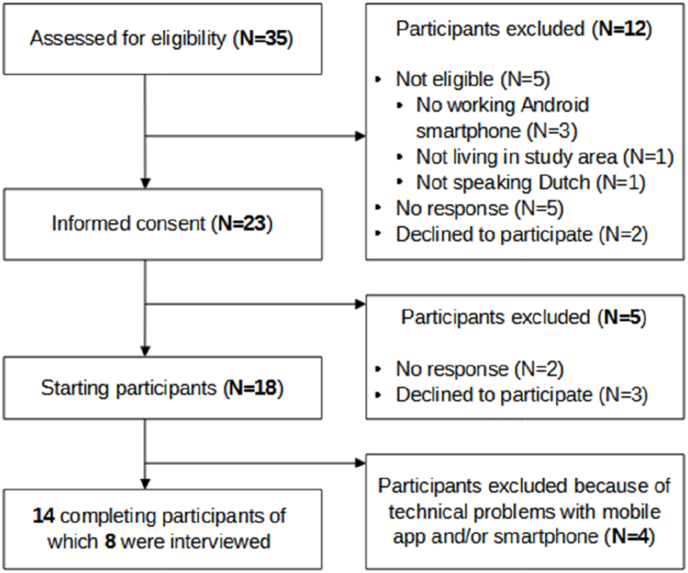
Flowchart of the study selection process.

Initially, 35 people who wanted to participate were assessed for eligibility (see the flowchart in Figure 2). After the exclusion of persons who were not eligible, did not respond, or declined to participate, 18 persons started in the study. Due to technical problems with the mobile app and/or their smartphone, 4 participants were excluded from participation. Eventually, 14 participants completed the study, of which 8 participants were interviewed. All 14 participants completed at least 5 d of the control week and 5 d of the intervention week. The 4 dropouts differed significantly regarding sex [*X*^*2*^ (1, *N* = 18) = 5.72, *P* = 0.04], but not regarding age (*z* = −1.77, *P* = 0.08)*,* overall self-reported health (z = −0.95, *P* = 0.34, and healthiness of the eating pattern (z = −0.59, *P* = 0.56). The proportion of males was higher in the dropouts compared with the 14 participants who completed the intervention.

See Table 2 for the descriptive statistics of the study sample (n = 14). Participants were mostly female (86%) and aged between 21 and 58 y. The education level was high for 11 participants and medium for 3 participants, and no participants had a low education level. The health literacy scores were categorized as problematic for 5 participants (36%) and inadequate for 1 participant (7%). Most participants chose the goal of healthier snacking (64%).

**TABLE 2:** Descriptive statistics of the study sample (n = 14)

Characteristic	Mean [SD] or n (%)
Sex	
Male, *n* (%)	2 (14)
Female, *n* (%)	12 (86)
Age in years, mean [SD] Education level, *n* (%)	27.3 [9.5]
High	11 (79)
Medium	3 (21)
Low	—
Health literacy, *mean* [SD]	12.9 [3.3]
Sufficient, *n* (%)	8 (57)
Problematic, *n* (%)	5 (36)
Inadequate, *n* (%)	1 (7)
Chosen goal, *n* (%)	
Healthier snacking	9 (64)
More vegetables and fruit	4 (29)
Less meat	1 (7)
Self-reported health, mean [SD]	3.3 [0.7]
Reasonable, *n* (%)	2 (14.2)
Good, *n* (%)	6 (42.9)
Very good, *n* (%)	6 (42.9)

NOTE. Low, medium and high education level are based on the categories of the International Standard Classification of Education [39]. Health literacy was assessed with the Dutch HLS-EU-Q16 [42].

#### Primary outcome: receptivity

Participants generally reported that they opened messages as soon as they saw them, but their engagement depended on the relevance of the content and the context of receipt. Although most participants expressed openness to receiving prompts, they emphasized that both timing and location played a crucial role in whether a prompt was perceived as useful or intrusive.

#### Contextual factors affecting receptivity

##### Location

Participants’ responses varied based on the setting in which prompts were received. In the app, 54 responses were collected for the question, “Are you open to prompts at this location?” with 53 answering “yes.” However, the questionnaire revealed that prompts were perceived as most useful at home (n = 13) and in supermarkets (n = 12), but less useful in settings like canteens (n = 4) and specialty stores (n = 3). Restaurants were particularly disliked as prompt locations (n = 11), as participants generally did not want to focus on health while dining out. Opinions were mixed for cafes, take-away outlets, and cafeterias.

Participants found prompts at home useful as they could consciously reflect on their eating habits and act on the suggestions, especially when healthy food was readily available. In supermarkets, prompts helped make healthier choices, but some participants noted that they often had a predetermined shopping list, reducing the impact of the prompt. One participant stated:

*“I consciously go to the supermarket with a plan, so prompts often did not translate into action.”*

Conversely, prompts in social settings like restaurants or cafes were often deemed irrelevant or even annoying. Participants described such contexts as times when they preferred to relax and indulge rather than be reminded of health goals. One participant remarked:

*“In places like a restaurant or café, you don’t go there to make a healthy choice.*

*You just want to have a nice moment and choose something you crave.”*

##### Timing

Timing also played a significant role in the perceived usefulness of prompts. Although the app collected 53 responses to the question, “Are you open to prompts at this moment?” with 51 answering “yes,” the questionnaire highlighted that the afternoon (n = 13) and evening (n = 8) were seen as the most suitable times. Morning prompts were less favored (n = 3), and many participants expressed a desire to receive prompts during the week rather than on weekends (n = 6).

Participants often preferred prompts in the afternoon and evening, as these were times when snacking or unplanned eating was more likely. Some also appreciated evening prompts with recipes or meal ideas, as they found these useful for planning future meals. A participant noted:

*“Those messages that came in the evening on recipes or something like that—those were helpful as they provided new ideas.”*

In contrast, weekend prompts were generally seen as intrusive, as participants preferred to enjoy more flexibility and indulgence. One participant expressed:

*“If you receive it [messages] on the weekend as well, I would think ‘not right now’ and then I would have an aversion to the app instead of it helping me.”*

##### Emotional factors associated with receptivity

Most participants did not see emotions as a primary factor associated with receptivity, but some patterns were observed. Participants were generally more open to receiving prompts when feeling calm (n = 8), stressed (n = 8), or happy (n = 4). However, they were less receptive when feeling angry (n = 9) or anxious (n = 4), as negative or intense emotions could hinder their ability to focus on health-related messages. Stress was particularly relevant, as some participants reported increased snacking when stressed and found prompts helpful as reminders to make healthier choices. In contrast, hurried or rushed states made prompts feel bothersome rather than supportive.

#### Exploratory outcomes

Results on feasibility will be presented according to the 3 main aspects: *1*) relevance and usability, *2*) perceived privacy, and *3*) perceived effectiveness.

##### Relevance and usability

Participants generally found the 3 available health goals relevant and achievable, as they were generic enough to fit a wide range of users’ needs. One participant remarked:

*“These goals are not very difficult to achieve because you can make small changes to snack healthier or eat less meat.”*

Healthier snacking was particularly valued for its daily relevance and alignment with increasing fruit and vegetable intake. However, some participants expressed a desire for broader goals that encompass a more holistic healthy lifestyle, including physical activity.

Overall, participants viewed the app’s personalized prompts as supportive of healthier food choices. Prompts delivered around meals and snacks were often perceived as well aligned with daily routines and helped raise awareness of everyday eating decisions. Location-based prompts were also positively received, as participants valued receiving support in food-related contexts. However, the perceived usefulness of these prompts varied depending on contextual factors, including the specific location, timing, and users’ emotional state.

Feedback on the app’s usability was mixed (Table 3). Although some participants found it easy to use, technical issues like high battery consumption and challenges with keeping the app activated negatively impacted the experience. Additionally, difficulties during installation, especially on certain Android versions, caused frustration. Users noted that keeping location settings enabled felt inconvenient, and the effort required to ensure the app was working correctly was seen as burdensome.

**TABLE 3:** Responses to postsurvey questionnaires (n = 14)

Question	Mean [SD] or n (%)
**1. Relevance and usability**	
Would you continue to use the app?	
Yes	1 (7.1)
Yes, but only when…	1 (7.1)
No	12 (85.7)
Would you install the app voluntarily?	
Yes	2 (14.3)
Maybe/Yes, but only when..	4 (28.6)
No	8 (57.1)
Would you recommend the app to others?	
Yes	4 (28.6)
Yes, but only when…	1 (7.1)
No	9 (64.3)
Would the app work better when it is more personalized?	
Yes	13 (92.8)
No	1 (7.1)
“The app is easy to install”	
Strongly agree	3 (21.4)
Agree	3 (21.4)
Neutral	3 (21.4)
Disagree	4 (28.6)
Strongly disagree	1 (7.1)
“The app is easy to use”	
Strongly agree	3 (21.4)
Agree	2 (14.3)
Neutral	4 (28.6)
Disagree	3 (21.4)
Strongly disagree	2 (14.3)
“The app fits within my daily routine”	
Agree	6 (42.9)
Neutral	4 (28.6)
Disagree	4 (28.6)
“The use of this app takes little time”	
Strongly agree	3 (21.4)
Agree	6 (42.9)
Neutral	1 (7.1)
Disagree	3 (21.4)
Strongly disagree	1 (7.1)
“The notifications were relevant”	
Agree	4 (28.6)
Neutral	4 (28.6)
Disagree	4 (28.6)
Strongly disagree	2 (14.3)
“I like the tone of the notifications”	
Strongly agree	2 (14.3)
Agree	6 (42.9)
Neutral	1 (7.1)
Disagree	5 (35.7)
“The number of messages generally was...”	
Way too little	1 (7.1)
Too little	3 (21.4)
Good	9 (64.3)
Too much	1 (7.1)
“My ideal number of prompts per day is...”	3.4 [1.2]
**3. Privacy concerns**	
“My privacy is not harmed with the use of this app”	
Strongly agree	3 (21.4)
Agree	3 (21.4)
Neutral	6 (42.9)
Disagree	2 (14.3)
**1. Perceived effectiveness**	
“My knowledge about healthy eating is broadened with the use of this app”	
Agree	1 (7.1)
Neutral	3 (21.4)
Disagree	5 (35.7)
Strongly disagree	5 (35.7)
“My choices of what I ate were more conscious with the use of this app”	
Strongly agree	1 (7.1)
Agree	1 (7.1)
Neutral	7 (50.0)
Disagree	5 (35.7)
Self-rated diet quality pre-study	2.9 [0.4]
Self-rated diet quality poststudy	3.03 [0.7]
Self-rated goal-related diet quality pre-study	2.1 [0.6]

NOTE. Participants rated their overall diet quality and goal-related diet quality (e.g., healthiness of snack intake) on a 5-point scale ranging from 1 (poor) to 5 (excellent).

The prompts themselves received varied feedback (Table 3). Although some users appreciated their positive and actionable tone, others found them repetitive or irrelevant when they did not match personal preferences or the availability of suggested products. The preferred number of prompts ranged from 2 to 6 per day (mean = 3.4). One participant appreciated the nonpushy tone, saying:

*“I thought that how it was formulated was very good and nice, without obligation and not pushy.”*

Despite the positive aspects, many participants indicated they would not continue using the app (Table 3). The reasons for this were mostly related to the limitations of the *EetWijzer* app. For instance, some participants could not reread received messages or did not receive many messages at all. On the contrary, other participants mentioned that they sometimes received an undesirable overload of messages when walking by or being at certain locations. Furthermore, some participants found the effort to integrate the app into daily routines too demanding, especially when friends or family were responsible for grocery shopping and cooking.

##### Perceived privacy

Participants expressed mixed opinions about the app’s impact on their privacy (Table 3). Despite these differences, a common theme emerged around the importance of transparency and control: participants emphasized the need for clear communication about the purpose of data sharing and the ability to decide what data to share and with whom. Overall, participants found it challenging to articulate specific reasons against sharing personal data.

Most participants indicated that they were comfortable with location tracking as long as they were informed, gave active consent, and did not consider the data highly personal. They felt that location sharing should be purposeful and beneficial, such as enhancing the relevance of prompts. As one participant noted:

*“As long as it works smoothly, the benefits [of location tracking] outweigh the burden.”*

Participants were generally open to sharing additional data types to receive personalized support, although preferences varied. Some were willing to share their calendar to receive timely prompts (e.g., after a meeting), whereas others questioned whether the benefits outweighed the effort of maintaining accurate calendar data. A more favored option was sharing a blocked calendar to conceal specific appointment details. Participants were also comfortable sharing data like nutritional intake, step count, body weight, and physical parameters such as stress, sleep, or heart rate, often tracked via smartphones or devices like Fitbit.

#### Perceived effectiveness

##### App effectiveness and impact on healthy eating

Most participants (n = 10) indicated that the app did not significantly increase their knowledge of healthy eating and did not lead to more conscious choices (n = 5) (Table 3). Additionally, most participants did not think that the app would help them reach their goals when used over a longer period (n = 8).

Despite the perceived limitations, quantitative analyses revealed positive changes in diet quality. Wilcoxon signed-rank tests showed significant improvements in self-rated diet quality (z = −2.22, *P* = 0.027) and goal-related diet quality (e.g., reduced meat consumption, healthier snacking, increased fruit and vegetable intake; z = −7.42, *P* < 0.001) from pre- to poststudy, indicating that participants rated their overall and goal-related diet quality higher after using the app (Table 3).

Interviews provided further context for these mixed findings. Participants reported that the app was particularly helpful in specific situations, such as when it encouraged healthier snack choices. For example, one participant reflected:

*“I received a message about snacking on vegetables, and examples like that made me think about how I could improve my life by snacking healthier.”*

Another participant appreciated the timing and relevance of prompts, stating: *“At certain moments, it definitely helped to receive a message that made me think. I was getting a little hungry, and if I hadn’t received that message, I might have eaten chocolate eggs. But because of the app, I chose something healthier.”*

##### Factors associated with perceived effectiveness

Perceived effectiveness varied considerably based on timing, location, and emotional state. Overall, 22.5% (n = 11) of the prompts were rated as helpful, 26.5% (n = 13) as neutral, and half (51%, n = 25) as not contributing to the chosen goal. Interview data echoed these results, indicating that prompt relevance was crucial for effectiveness. Participants noted that when suggestions aligned with available options and current needs, they were more likely to act on them.

A mixed model analysis explored whether openness to location, time, and emotion was associated with perceived effectiveness. No significant associations were found for openness to location (F = 0.17; *P* = 0.69) or time (F = 0.67; *P* = 0.42). However, emotion was significantly associated with effectiveness (F = 2.53; *P* = 0.045). Prompts were most effective when participants felt happy and least effective when feeling sad, surprised, or disgusted (Table 4).

**TABLE 4:** Frequencies and mean of perceived effectiveness scores per emotional state

Emotion	Very unlikely/ unlikely PE	Neutral PE	Likely/very likely PE	Mean and SD of PE score	Number of responses
Happy	30.0%	20.0%	50%	3.20 (1.23)	10
Relaxed	56.7%	26.7%	16.6%	2.30 (1.18)	30
Stressed	33.3%	50%	16.7%	2.83 (0.75)	6
Sad	100%	—	—	2.0 (2.0)	1
Surprised	100%	—	—	2.0 (2.0)	1
Disgusted	100%	—	—	1.0 (1.0)	1

Abbreviation: PE, perceived effectiveness of prompt.

#### Differences between educational and health literacy levels

Lower-educated participants liked the tone of the prompts less than their higher-educated counterparts (z = −2.25, *P* = 0.03) and found the app more challenging to install (z = −2.31, *P* = 0.02). Regarding perceived effectiveness, there were no differences between groups in knowledge expansion. However, participants with higher educational levels reported making more conscious food choices as a result of using the app compared with those with lower educational levels (z = −2.3, *P* = 0.021). No significant differences were found between groups concerning ease of use, relevance, the time required to use the app, or how well it fits into their daily routines.

When comparing participants with different levels of health literacy, no significant differences were found across any of the measured variables.

## Discussion

This study explored user receptivity to the *EetWijzer* app, a researcher-developed smartphone-based intervention providing JIT support for healthier food choices. By combining in-app responses with interviews in real-world contexts, this study provided insight into when and where users are receptive to receiving support for healthy eating. This is important, since receptivity has emerged as a critical determinant of effectiveness [48].

Participants were generally receptive to the core elements of the app, including location and time-based prompts. In-app data showed high openness to receiving prompts across contexts, whereas questionnaire and interview findings indicated a preference for receiving prompts at home or in the supermarket, where participants were more likely to use their phones, such as for grocery lists. Similarly, previous research found that interventions are generally more appreciated and are more likely to be responded to during daytime, but not at work, and if the participant is already holding their phone [49]. Participants also noted that a lack of healthy options in their environment made it difficult to act on the app’s prompts. Similarly, Terzimehić et al. [17] found that participants ate unhealthier when they lacked healthy alternatives nearby or were unaware of them. This suggests the need to provide support earlier in the decision-making process, such as during grocery planning or displaying nearby healthy food options in unfamiliar areas before going there. One promising direction is to analyze the users’ behavior patterns to identify recurring situations in which support is needed for making healthy choices [17].

The exploratory feasibility findings indicated that participants generally found the health goals and prompts of the *EetWijzer* app relevant and useful. However, participants emphasized the importance of having control over timing, location, frequency, and type of prompts received. These findings align with previous research suggesting that tailored features increase engagement [50] and prevent prompts from being perceived as unwanted during food shopping [51]. Experts similarly highlighted that consumers should have autonomy in deciding what type of support they want and what data they are willing to share [51].

The usability of the app received mixed ratings due to bugs and high battery drain. These technical challenges are critical, as they can undermine user engagement and the overall feasibility of the app [52]. Addressing these issues will be essential in future iterations to ensure that users remain engaged and that the app delivers intended support effectively.

Participants were generally comfortable sharing their data, such as GPS location, in exchange for more personalized experiences. This finding aligns with broader mHealth research showing that the perceived benefits of personalization often outweigh privacy concerns and shape users’ willingness to disclose personal information [12,[53], [54], [55]].

Although participants did not always recognize the app’s direct impact on achieving their dietary goals, self-rated diet quality and goal-related behaviors improved over the study period. These changes may have been driven by increased awareness of healthy food choices, which participants reported as having improved throughout the study.

Participants generally preferred receiving prompts during positive emotional states rather than during stress or negative emotions. At the same time, moments of stress may represent important opportunities for intervention, given their association with unhealthy eating behaviors [55,56]. This highlights a broader challenge in designing JIT interventions: balancing user receptivity and comfort with delivering support during moments that are most relevant for behavior change. Similar tensions have been reported in other JIT intervention studies [12].

Although some studies suggest that apps are an acceptable means for supporting healthier food behaviors across socioeconomic groups [56], the current study identified differences in receptivity, usability, and perceived effectiveness between participants with lower and higher educational levels. No significant differences were observed across health literacy levels. This is notable given that previous literature has identified health literacy as a critical determinant linking socioeconomic position to health disparities [57]. One possible explanation for our finding is the limited variation in health literacy scores within our sample, which may have reduced the ability to detect differences. The findings of the present study highlight the importance of considering educational level and broader contextual demands [[58], [59], [60]] when designing inclusive digital health interventions.

Overall, the findings underscore the importance of personalization, as receptivity appeared to dependent on contextual factors such as location, timing, and emotional state. Future work could explore machine learning techniques to predict unhealthy behaviors and “risky” moments and environments where unhealthy behavior is likely to occur [61] and increase long-term app engagement [38,50]. Reinforcement learning algorithms can be used to continuously adapt the intervention and optimize intervention delivery and or content based on the behavior of the individual and their context. However, understanding which contextual factors are most relevant for personalization remains an important first step in developing feasible and scalable adaptive interventions [38].

### Strengths and limitations

A strength of this study is the use of both quantitative and qualitative data collection methods. Next to pre- and poststudy surveys, in-app data were gathered immediately following each prompt, increasing ecological validity and reducing recall bias. Several limitations should also be acknowledged. First, the relatively small sample size may limit the generalizability of the findings. Second, the interview results might not fully capture the perceptions of all participants, particularly those who declined to be interviewed. Third, the assessment of health literacy was introduced post hoc and was only measured at follow-up, which means the scores could have been influenced by study participation. Fourth, the study was limited to individuals who owned Android devices, limiting generalizability to other operating systems. Lastly, we used a manual location mapping approach during development to define the locations and their notification radius. This process may have introduced slight errors in the boundaries between outlet types, and proximity detection may have been affected by the quality of GPS signals and physical obstructions (e.g., buildings or dense areas). However, for this feasibility study, this approach provided a simple and practical way to gather initial data on user receptivity to location-based prompts in a small study area.

### Implications and recommendations

The study’s findings have several important implications for the design of JIT-based health interventions. First, interventions should account for variability in contextual receptivity, particularly location, emotional state, and decision-making opportunity. Second, transparency and user control over data sharing are essential for acceptability. Third, the study emphasizes the importance of striking a balance between what users perceive as effective and what they truly need to change their behavior meaningfully. Interventions should respect user comfort and preferences yet also be designed to gently challenge these preferences when necessary to promote deeper and more sustained behavior change. Fourth, technical reliability is a prerequisite for successful behavioral intervention delivery. Finally, there is a need for more inclusive research that actively involves participants from diverse socioeconomic backgrounds to ensure these interventions are usable, useful, and effective across different population groups.

In conclusion, this mixed-methods study demonstrates that receptivity to JIT support for healthy eating is context-dependent, influenced by location, timing, and emotional state. Participants showed higher receptivity for prompts at home, at supermarkets, during weekdays, and when they are in a positive or calmer emotional state. Furthermore, the findings suggest that the effectiveness of such interventions depends on balancing user comfort and autonomy with delivery at behaviorally relevant moments. Together, these results provide initial guidance for the design of more adaptive and personalized digital dietary interventions.

## Data availability

Data described in the manuscript, code book, and analytic code are made publicly and freely available without restriction at https://data.4tu.nl/datasets/bac66c61-83c2-4d4a-8770-19b9753ca770.

## Declaration of Generative AI and AI-assisted Technologies in the Writing Process

During the preparation of this work, the author(s) used ChatGPT to assist with rewriting and phrasing certain sentences. After using this tool, the authors reviewed and edited all content as needed and take full responsibility for the content of the publication.

## Funding

This publication is part of the project “Measuring, analyzing and mapping environmental influences on (and changes in) spatial patterns: evidence for Just-in-Time adaptive interventions” with project number 555003027 of the research program “E-health” which is financed by the Dutch Research Council (ZonMw).

LW’ contribution was partly funded by the Dutch Research Council (NWO), grant number 452-14-014, and partly by the 4 Dutch Technical Universities, 4TU—RECENTRE program (4TU-UIT-468).

AS’ contribution is funded by mVital@2040 project funded by Wageningen University & Research (mVital@2040 project, which is a part of the Vitality Academy and connected to the EWUU Alliance (Eindhoven University of Technology, Wageningen University & Research, Utrecht University, and University Medical Centre Utrecht)).

MS’ contribution was partly funded by the 4 Dutch Technical Universities, 4TU—Pride and Prejudice program (4TU-UIT-346).

## Conflicts of interest

The authors report no conflicts of interest.
