# Supplementary material for: Exploring the Receptivity and Feasibility of Just-In-Time Support for Healthy Food Choices: Mixed-Method Insights for Adaptive Intervention Development
Source: Curr Dev Nutr. 2026 Jun 6;10(7):109391. doi: 10.1016/j.cdnut.2026.109391 (PMC13324450; doi:10.1016/j.cdnut.2026.109391)
Supplement: multimedia component 1 [file mmc1.docx]

**Supplementary Table 1. Example prompts delivered to participants.**

| **Goal** | **Context** | **Example prompts** |
| --- | --- | --- |
| More fruit and vegetables | Near a supermarket (16:00-20:00) | Make sure to eat 250 grams of vegetables per person tonight.  Soup is an easy way to get lots of vegetables; try this spinach soup. |
| Less meat intake | Near a restaurant or lunchroom | Choose the vegetarian option, for example a vegetarian burger or a vegetable curry.  Stick to your goal tonight! Choose the vegetarian option. |
| Healthier snacking | Near a pub | Alternate your drinks with water  Choose an alcohol-free drink. |

*Note.* Prompts were tailored to the selected goal, food outlet, and time of day.
