# Supplementary material for: Exploring the Receptivity and Feasibility of Just-In-Time Support for Healthy Food Choices: Mixed-Method Insights for Adaptive Intervention Development
Source: Curr Dev Nutr. 2026 Jun 6;10(7):109391. doi: 10.1016/j.cdnut.2026.109391 (PMC13324450; doi:10.1016/j.cdnut.2026.109391)
Supplement: multimedia component 2 [file mmc2.docx]

**Supplementary Methods 1.** Questionnaires.

1. **Pre-study questionnaire.** Participant characteristics and baseline measures.

| **Question** | **Response options** |
| --- | --- |
| What is your age? | Open-ended |
| What is your gender? | Man / Woman / Other / I would rather not say |
| What is your highest attained level of education? | Primary education; Pre-vocational secondary education; Senior general secondary education (first three years); Pre-university secondary education; Assistant training; Basic vocational training; Vocational training; Middle management or specialist education; Higher education; Bachelor’s degree; Master’s degree; Doctoral degree |
| Do you want to improve your eating pattern? | Yes / No / Other |
| Do you have trouble finding, understanding, and using health information? | Yes, always / Yes, sometimes / No, never |
| How would you describe your health? | Excellent / Very good / Good / Fair / Poor |
| How healthy is your eating pattern in general? | Excellent / Very good / Good / Fair / Poor |
| Which goal would you like to work on during this study? | Healthier snacking / Eating less meat / Eating more vegetables and fruit |

**Goal-specific questions** (asked depending on selected goal)

| **Question** | **Response options** |
| --- | --- |
| How healthy is your intake of snacks / meat / vegetables and fruit in general? | Excellent / Very good / Good / Fair / Poor |
| Can you briefly explain this? | Open-ended |

**B. Post-study questionnaire.** Experience and perceived impact.

| **Question** | **Response options** |
| --- | --- |
| How did you come across this research? (e.g., Facebook message, supermarket flyer) | Open-ended |
| How healthy was your eating pattern in the past week (during app use)? | Excellent / Very good / Good / Fair / Poor |
| My chosen goal was: | Healthier snacking / Eating less meat / Eating more vegetables and fruit |

**Goal-specific evaluation** (asked depending on selected goal)

|  |  |
| --- | --- |
| How healthy was your intake of snacks / meat / vegetables and fruit in the past week? | Excellent / Very good / Good / Fair / Poor |

|  |  |
| --- | --- |
| Using the app contributed to achieving my goal. | Strongly disagree – Strongly agree (5-point Likert scale) |
| In what way did the app (not) contribute to achieving your goal? | Open-ended |
| Do you think you can achieve your goal if you used the app for a longer period of time? | Yes / No / Other |

**Contextual preferences for message delivery** (Multiple answers possible)

**Locations where messages are most useful**

- Supermarket
- Restaurant
- Café
- Specialist shop (e.g., butcher, bakery, fish shop)
- Takeaway (e.g., pizza, sushi)
- Canteen
- Snack bar
- At home

**Locations where messages are not desired**

- Same options as above

**Timing preferences**

| At which moments is receiving messages most useful for you? | Morning / Afternoon / Evening / Night / Other |
| --- | --- |
| At which moments do you not want to receive messages? | Morning / Afternoon / Evening / Night / Other |
| On which days are messages most useful? | Weekdays / Weekends / All week / Other |
| On which days do you not want to receive messages? | Weekdays / Weekends / All week / Other |

**Emotional context** (Multiple answers possible)

| When feeling which emotions is receiving messages most useful? | Happy / Calm / Stressed / Anxious / Sad / Surprised / Angry / Disgusted / Other |
| --- | --- |
| When feeling which emotions do you not want to receive messages? | Same options as above |

**Content and frequency preferences**

| My preference is to receive: | Recipes / Restaurant menu options / Tips for eating healthier / Reminders of my goal / Other |
| --- | --- |
| The number of messages generally was: | Way too little / Too little / Good / Too much / Way too much |
| For me, the best number of messages per day is: | 1 / 2 / 3 / … / 20 |
| If the app takes my personal preferences into account, do you think it would work better for you? | Yes / No / Other |

**App evaluation statements**
(5-point Likert scale: Strongly disagree – Strongly agree)

- I like the tone of the notifications
- The notifications I received were relevant
- My knowledge about healthy eating increased through use of the app
- My food choices became more conscious through use of the app
- The app fits within my daily routine
- The app is easy to install
- The app is easy to use
- Using the app takes little time
- My privacy is not compromised by using this app

**Future use**

| Would you continue to use the app after this study? | Yes / No / Other |
| --- | --- |
| Would you install the app if participation were not required? | Yes / No / Other |
| Would you recommend this app to others? | Yes / No / Other |
| Do you have any additional comments about the app? | Open-ended |
| May we contact you for a short telephone interview (±20 minutes)? | Yes / No |

**C. In-App Questions (After each prompt)**

| Are you open to receiving this message at this location? | Yes / No |
| --- | --- |
| Are you open to receiving this message at this moment? | Yes / No |
| How are you feeling at this moment? | Happy / Calm / Stressed / Anxious / Sad / Surprised / Angry / Disgusted |
| This message contributes to my goal (healthier snacking / eating less meat / eating more vegetables and fruit). | Very unlikely / Unlikely / Neutral / Likely / Very likely |

**D. In-App questions (End of day)**

| **Question** | **Response options** |
| --- | --- |
| The number of messages today was: | Way too much / Too much / Good / Too little / Way too little |
| Was there a time today when you wished you had received a message but did not? If so, when and where? | Open-ended |
| Which messages did you like today and why? | Open-ended |
| Which messages did you not like today and why? | Open-ended |
| Did you notice anything today that could be improved in the app? | Open-ended |
