# Supplementary material for: Exploring the Receptivity and Feasibility of Just-In-Time Support for Healthy Food Choices: Mixed-Method Insights for Adaptive Intervention Development
Source: Curr Dev Nutr. 2026 Jun 6;10(7):109391. doi: 10.1016/j.cdnut.2026.109391 (PMC13324450; doi:10.1016/j.cdnut.2026.109391)
Supplement: multimedia component 3 [file mmc3.docx]

**Supplementary Methods 2. Interview Guide**

**Introduction**

Thank you for participating in this online interview. The purpose of this interview is to better understand your experience using the *EetWijzer* app. With your permission, the conversation will be recorded. You may turn off your camera at any time if you prefer.

This study investigates an app that sends goal-related messages based on time and location (e.g., in a supermarket or around lunchtime), aiming to support food choices at relevant moments.

**Interview questions**

1. How useful do you think an app that sends messages at specific times and locations can be for you? Why?
2. How easy was the app to use?
3. If the app did not always work properly, how easy was it to use when it did work as intended?
4. You indicated that you did not want to receive messages at certain moments, days, or locations.
   - Why did you (not) want to receive messages in these situations?
5. Were there times or locations when you did not receive messages but would have liked to?
6. What were your reasons for not using the app at certain times or locations?
7. Are there specific emotions or feelings that make you more or less open to receiving messages? Why?
8. The app uses your location to determine which messages to send.
   - What are your thoughts on the use of this personal data (e.g., privacy)?
9. What types of personal data would you be willing to share to make the app more supportive?
   *Examples (if needed): calendar data, stress indicators, dietary intake, weight, glucose levels, pedometer data.*
10. During the second week of the study, how did you interact with the messages you received?
    - Did you open them immediately or later?
    - What did you do with the information?
11. How did you experience the messages overall?
12. What kinds of messages would you like to receive in the future?
13. You indicated that the app did (or did not) contribute to achieving your goal.
    - In what way?
    - If applicable, what changed in your eating behavior or thinking?
14. Do you think the app influenced your food-related movement patterns (e.g., visiting different stores or going shopping less often)? Why?
15. Why would you (or would you not) continue using this app after the study?

**Closing**

These were all the questions I had for you. Thank you very much for your time and insights. Do you have any additional comments or questions about the study?
